# Recurrent Gastrointestinal Stromal Tumor With Chondroid Differentiation After Imatinib Therapy: An Unusual Case and Literature Review

**DOI:** 10.7759/cureus.54842

**Published:** 2024-02-24

**Authors:** Patricia Le, FNU Monika, Ahmed Sabri, Joyce Kovar, Nicholas Dietz

**Affiliations:** 1 Pathology, Creighton University School of Medicine, Omaha, USA

**Keywords:** molecular sequencing, immunohistochemistry staining, gastrointestinal pathology, imatinib therapy, gastrointestinal stromal tumor (gist)

## Abstract

Gastrointestinal stromal tumor (GIST) is the most common mesenchymal tumor in the gastrointestinal tract and is most commonly seen in the stomach. The standard treatment for patients with advanced GISTs include both surgical resection and imatinib therapy. There have been cases that document the alterations of patients' GIST histomorphology both with primary GIST prior to imatinib therapy and with recurrent GIST after imatinib therapy. However, there has been no documented case of a patient who has recurrent GIST with chondroid differentiation at the primary site after imatinib therapy.

In this article, we report an incidental finding of a 58-year-old patient who had two treatments of imatinib therapy prior to surgical resection of her recurrent GIST in her stomach. We also explore through a mini-literature review the various cases of GIST with chondroid differentiation that have been reported to compare the histomorphology, immunophenotype, and patient demographic of these cases. This article is significant for reporting a rare finding of GIST after imatinib therapy and highlights the various presentations that GIST could acquire after imatinib therapy that exclude another malignant process, such as chondrosarcoma.

## Introduction

Gastrointestinal stromal tumors (GISTs) are most frequently seen in the stomach (80%) and are the most common mesenchymal neoplasm in the gastrointestinal tract [1]. The most common histopathologic presentations of GIST include spindle cell type (70%), epithelioid cell type (20%), and mixed type (10%) [2,3]. The standard treatment of GIST includes surgical resection, and for more advanced tumors, either or both preoperative and postoperative imatinib therapy is administered [4].

There have been reports of changes in the histomorphologic features of GIST after imatinib therapy, both at the primary site and at the site of metastasis. Pauwels et al. described a case of a 46-year-old male patient with primary spindle-cell type GIST at the stomach that has metastasized to the liver. After imatinib therapy, there was a tubulopapillary and epithelioid growth pattern found in the liver [5]. There was another case described by Pulvers et al. of a 54-year-old female who had metastasized GIST to the liver and was found to have chondroid metaplasia in the liver at the site of the GIST post-imatinib therapy [6].

However, there has been no report of GIST with chondroid differentiation in a primary tumor site after imatinib therapy. This case highlights a unique presentation of GIST with chondroid differentiation after imatinib therapy. Furthermore, we provide a literature review of the documented cases of GIST with chondroid differentiation pre- and post-imatinib therapy to explore other possible ways cartilage is identified in GIST.

This case was previously presented as a meeting abstract at the 2023 American Society of Clinical Pathology Conference on October 18 and 19, 2023.

## Case presentation

**Figure 1:**
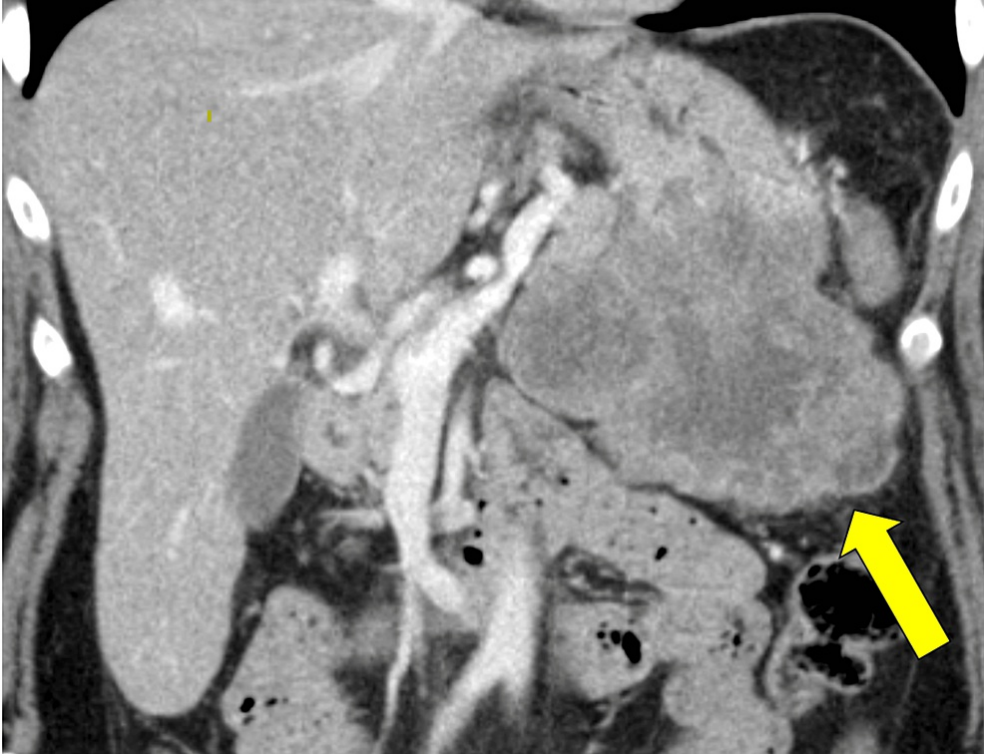
CT Abdomen of Primary Gastrointestinal Stromal Tumor (GIST) in Posterior Wall and Greater Curvature of Stomach

A 58-year-old female originally presented to the Emergency Department at Creighton University Medical Center-Bergan Mercy due to a syncopal episode and was incidentally found to have an 11 x 8.5 x 7 centimeters (cm) mass arising from the posterior wall and greater curvature of stomach on computed tomography scan (CT) of the abdomen (Figure 1).

**Figure 2:**
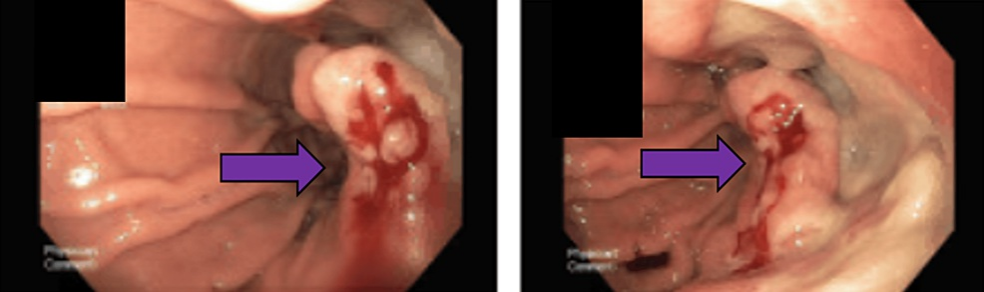
Mass in Gastric Body Found in Endoscopy Image

Endoscopy was performed and gastroenterologists identified a large, fungating and ulcerated mass in the gastric body that was biopsied (Figure 2).

**Figure 3:**
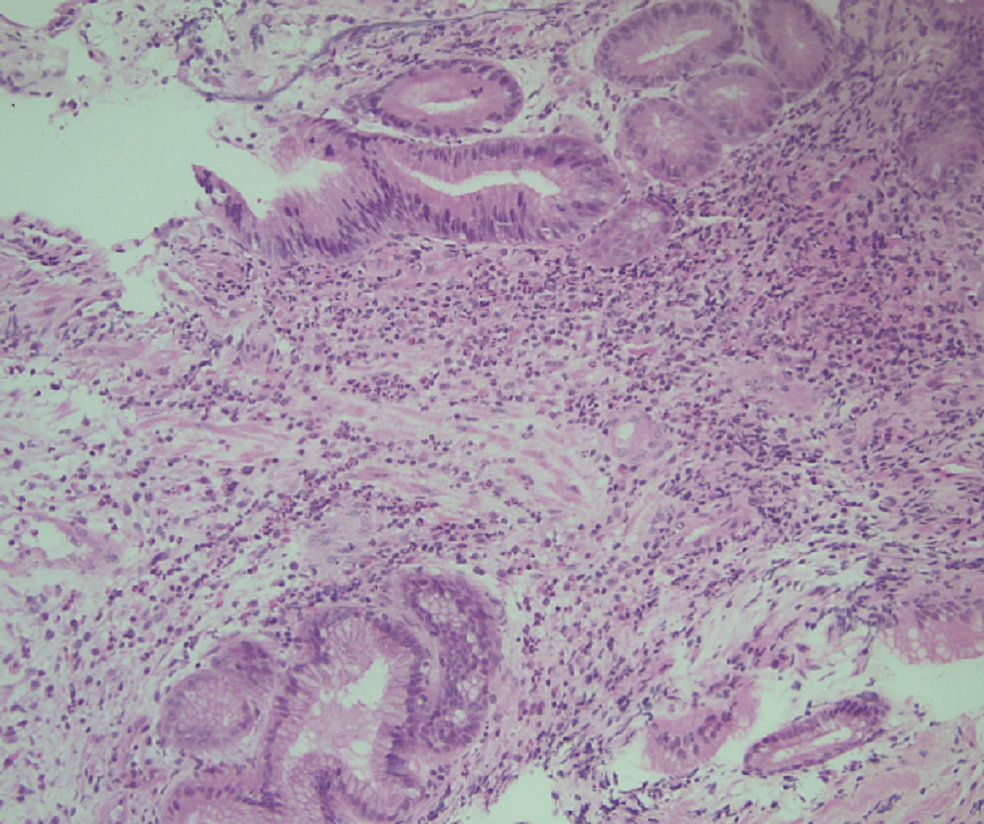
Hematoxylin and Eosin Stain of Needle Biopsy of Gastric Body Mass at 200x Magnification. Spindle cell Morphology is Identified in the Lamina Propria.

**Figure 4:**
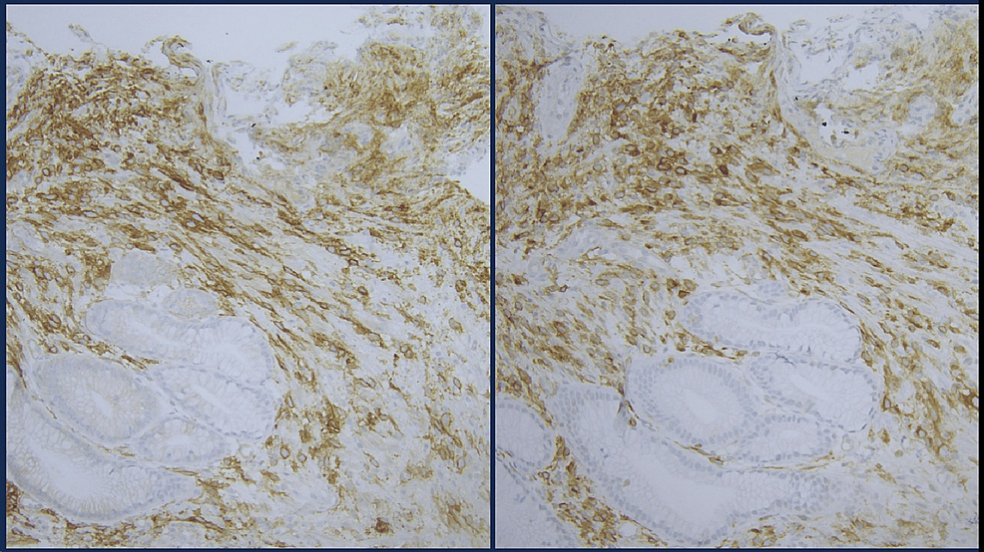
Immunohistochemical Stains DOG1 (Left) and CD117 (Right) of Needle Biopsy of Gastric Body Mass at 200x magnification. DOG1 and CD117 Both Highlight Spindle Cell Lesion.

The initial biopsy of the gastric mass showed a spindle cell morphology and stained positively for DOG1 and CD117 (Figures 3, 4). The patient subsequently underwent a partial gastrectomy. 

**Figure 5:**
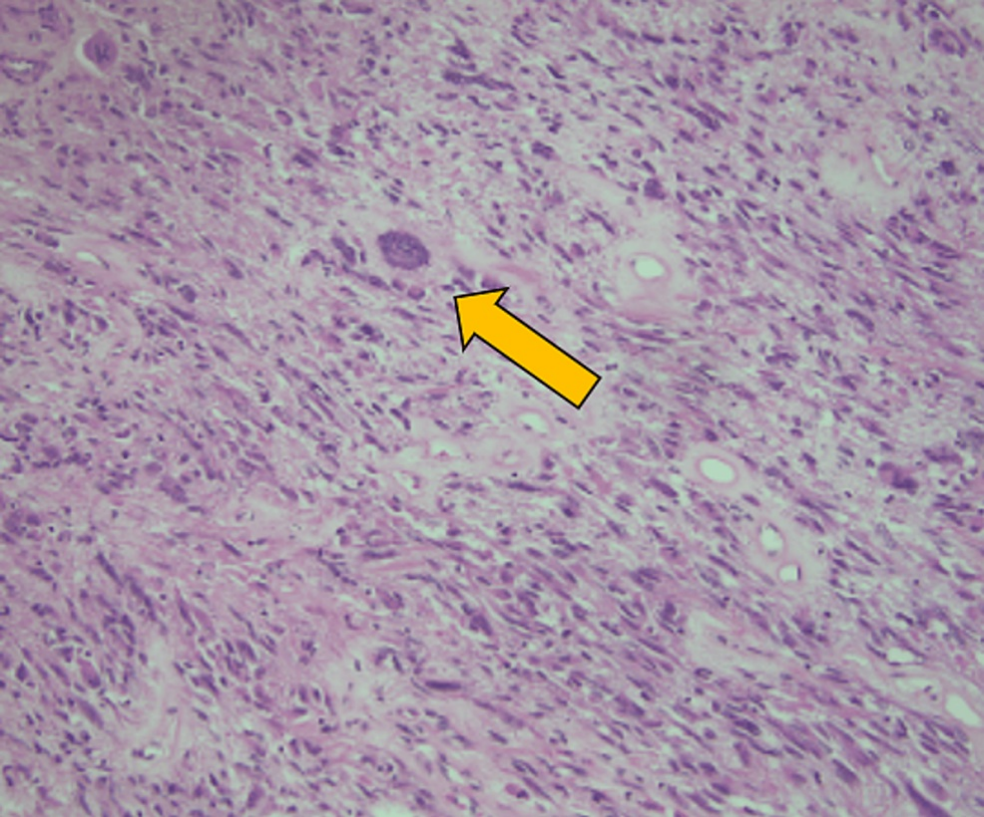
Hematoxylin and Eosin Stain of Section from Initial Partial Gastrectomy of Gastrointestinal Stromal Tumor (GIST) on 200x Magnification. Orange Arrow Points at Focal Bizarre Epithelioid Cell in a Background of High-Grade Spindle Cell Lesion.

Grossly, her initial partial gastrectomy showed a bulging subserosal multilobular tan to reddish-gray mass measuring 13.0 x 9.5 x 6.4 cm. Cut surfaces ranged from solid tan fibrous to vaguely whorled and trabeculated. The pathology of the initial gastrectomy found high-grade spindle cell type GIST with focal bizarre epithelioid cells (Figure 5).

**Figure 6:**
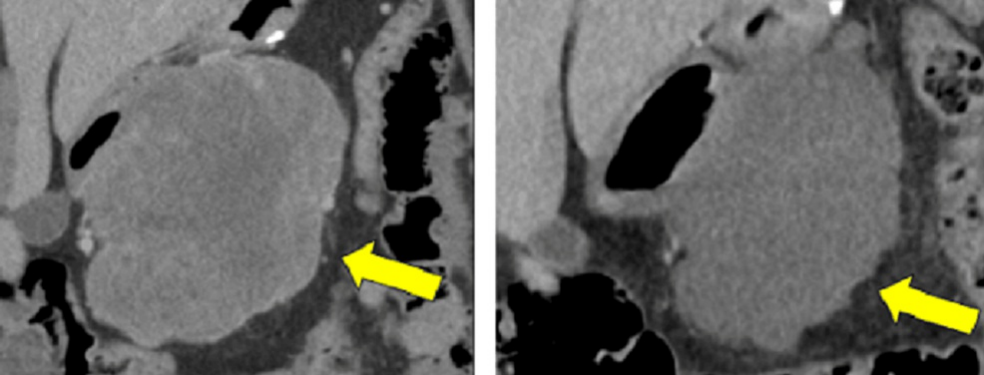
CT Abdomen of Recurrent Gastrointestinal Stromal Tumor (GIST) Before (Left) and After (Right) Second Course of Imatinib Therapy

**Figure 7:**
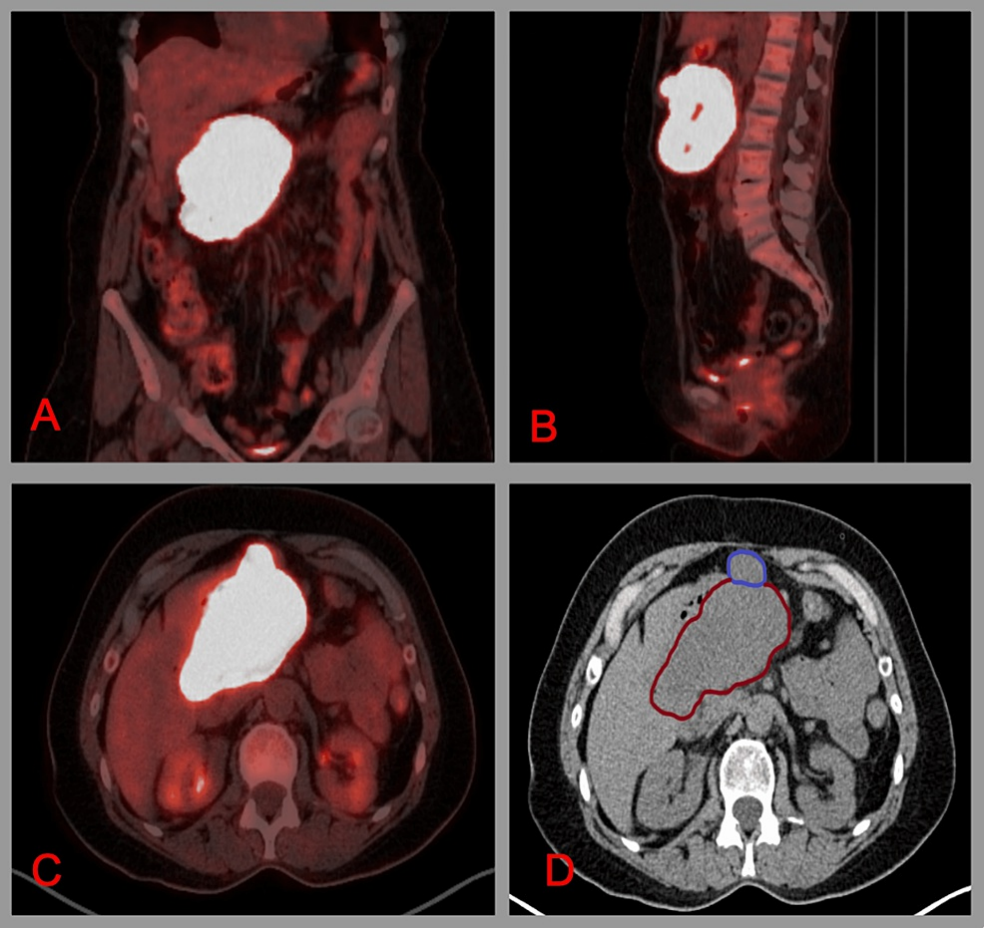
Positron Emission Tomography-Computed Tomography in Coronal (A), Sagittal (B), and Axial (C) Planes. Corresponding Computed Tomography of Abdomen with Red Outline Identifying Mesenteric Mass and Blue Outline Identifying Lymph Node (D).

The patient was subsequently treated with imatinib for three years. During her annual CT surveillance imaging two years after imatinib therapy, she was found to have a heterogeneous, centrally necrotic lobulated mass in the central abdomen that abuts the stomach measuring 11.8 x 11.7 x 6.7 cm, compatible with localized recurrence (Figure 6). Positron Emission Tomography-Computer Tomography also found a heterogeneously hypermetabolic mass from the body and greater curvature of the stomach with necrotic areas measuring 11.6 x 10.9 x 7.1 cm with dominant metabolic activity in the right inferior aspect of the mass (Figure 7). Additionally, there were hypermetabolic nodes anterior to the mass that were suspicious for metastasis.

**Figure 8:**
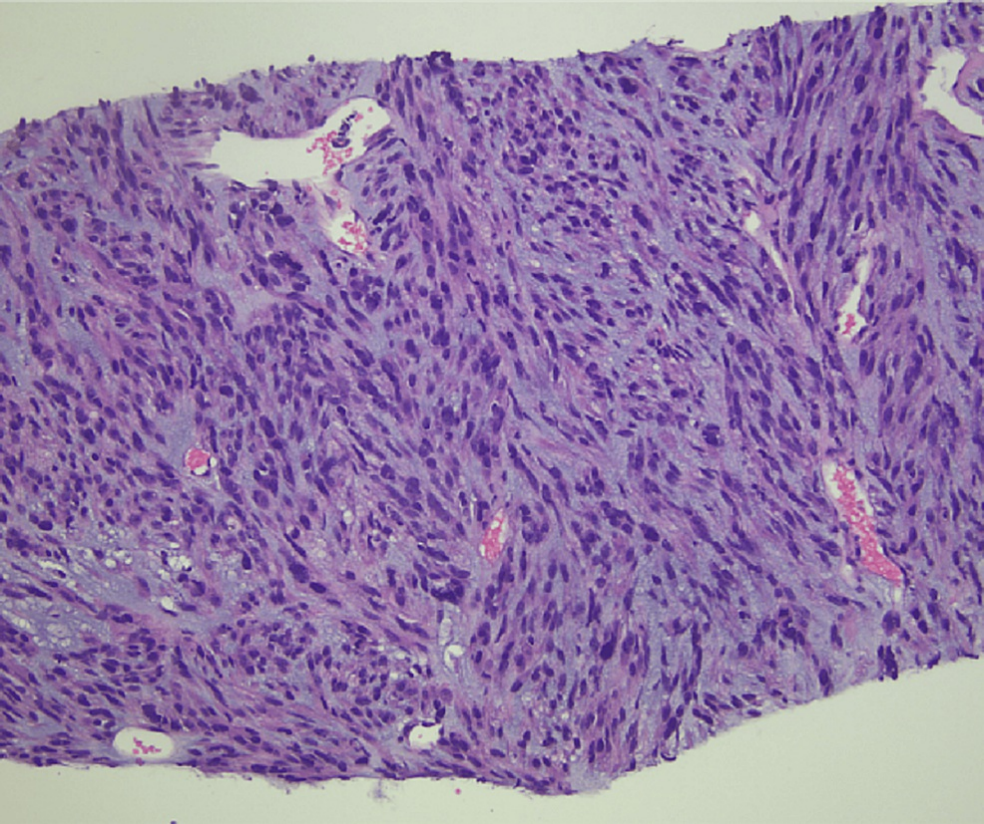
Hematoxylin and Eosin Stain of Needle Biopsy of Right Mesenteric Mass on 200x Magnification. Tissue with Spindle Cell Morphology is Present.

**Figure 9:**
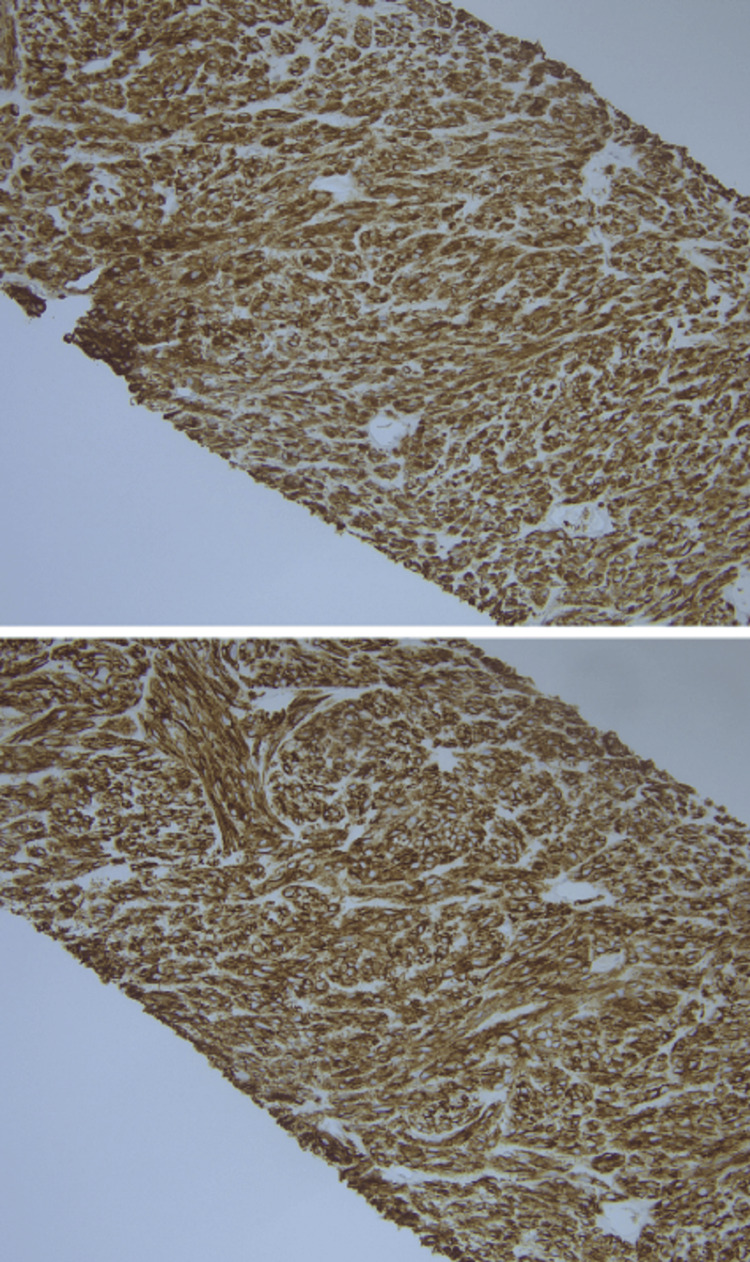
Immunohistochemical Stains CD117 (Top) and DOG1 (Bottom) of Right Mesenteric Mass at 200x Magnification.

The pathology from the biopsy of the right mesenteric mass showed spindle cell morphology on hematoxylin and eosin stain (Figure 8) and was positive for DOG1 and CD117 on immunohistochemistry (Figure 9). The patient subsequently underwent another partial gastrectomy. The mass measured 10.4 x 9.1 x 4.4 cm and had a heterogenous cut surface with tan-gray at the periphery and tan-yellow necrosis in the center. Additionally, the tumor appears to have invaded the adjacent adipose tissue.

**Figure 10:**
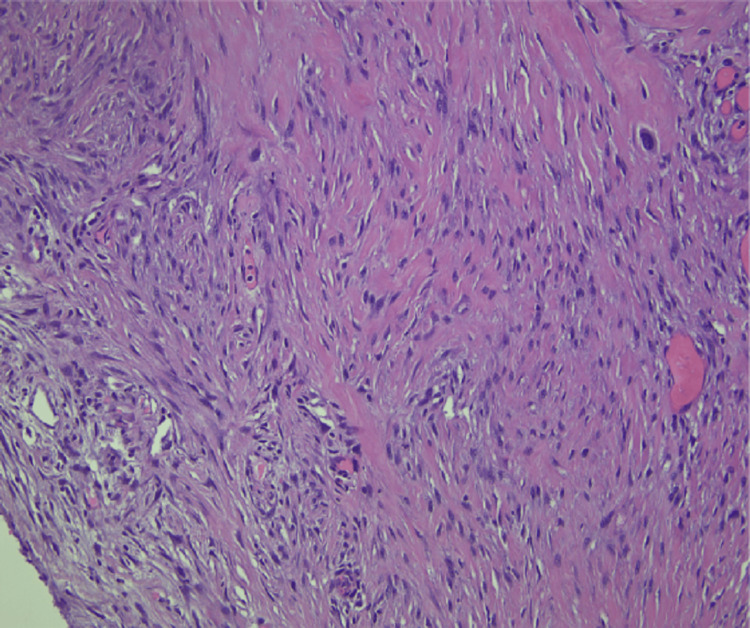
Hematoxylin and Eosin Stain of Partial Gastrectomy for Recurrent Gastrointestinal Stromal Tumor (GIST) at 200x Magnification. Tissue Section Shows Spindle-Cell Morphology.

**Figure 11:**
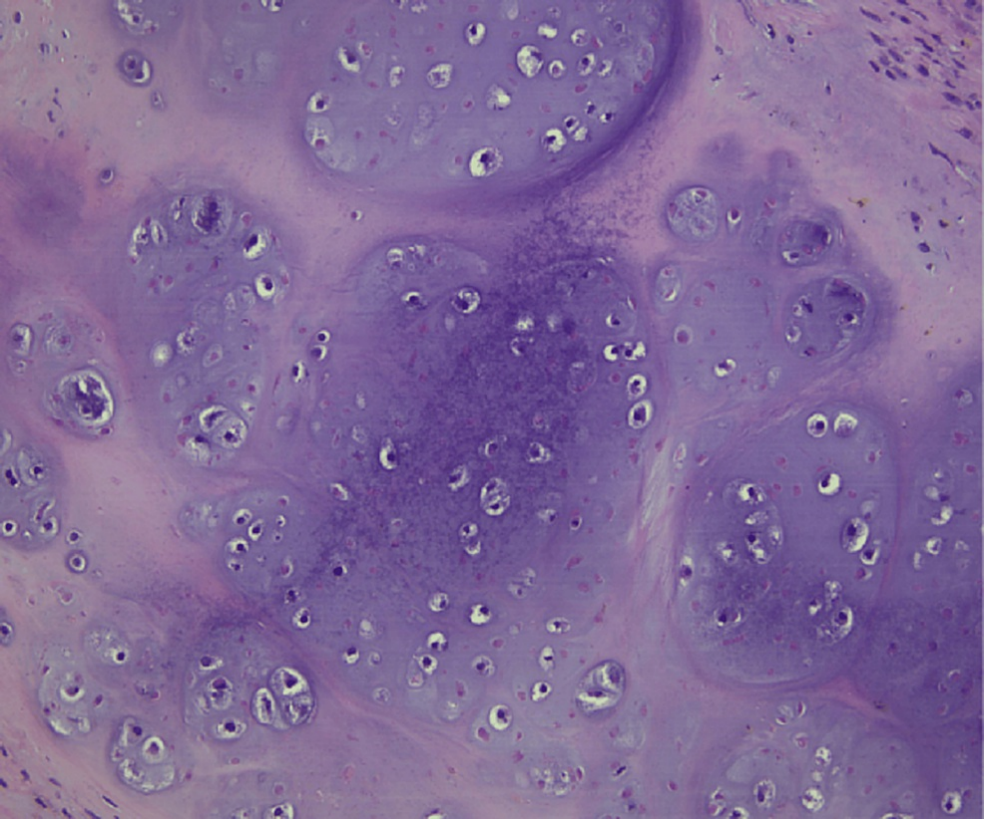
Hematoxylin and Eosin Stain of Partial Gastrectomy for Recurrent Gastrointestinal Stromal Tumor (GIST) at 200x Magnification. Tissue Section Shows Chondroid Differentiation.

The tissue sections showed high grade spindle-cell morphology with chondroid differentiation (Figures 10, 11). After discovery of recurrent GIST, the patient was restarted on imatinib therapy. Table 1 outlines pertinent information in the pathology reports pertaining to our patient’s GIST diagnosis.

**Table 1:** Pertinent Pathology Information of Patient’s Primary, Metastatic, and Recurred Gastrointestinal Stromal Tumor (GIST) HPF=High-Power Field

Year	Procedure/Specimen	Histomorphology	Immunohistochemistry/Molecular and Cytogenetics	Additional Findings
2016	Biopsy/Gastric Mass	Spindle cell type	Focally DOG1 (+), CD117 (+), Desmin (+), S100 (+), Cytokeratin AE1/AE3 (-), Low Molecular Weight Cytokeratin (-), High Molecular Weight Cytokeratin (-), CD34 (-), MelanA (-)	
2016	Partial Gastrectomy/Stomach Mass from Greater Curvature of Stomach	Spindle cell type with focal bizarre epithelioid cells and necrosis and mitotic rate of 23/50 HPF	No immunohistochemistry or molecular/cytogenetics was performed	Histologic grade G2, High Grade and Pathologic Stage pT4
2022	Needle biopsy/Right mesenteric mass	Spindle cell type	DOG1 (+), CD117 (+) Ki-67 >30% with *c-KIT *mutation on exon 11 and negative for *PDGFRα *mutation	
2023	Partial gastrectomy/Stomach Mass from Greater Curvature of Stomach	Spindle cell type with focal chondroid differentiation	Spindle cells: DOG (+), CD117 (-) in neoplastic cells and (+) in background cells and chondroid regions: DOG (-), CD117 (-)	Histologic grade G2, High Grade and Pathologic Stage pT4

For processing of the tissue, the tissue was immersed in 10% formalin for fixation. The selected sections were then dehydrated, paraffin embedded, and cut into 4 μm sections for hematoxylin and eosin stain.

For immunohistochemical staining, the laboratory uses UltraView© or OptiView© detection kit with a known positive control. All antibodies except anti-CD117 were purchased from Ventana (Oro Valley, AZ, USA). Anti-CD117 was purchased from Cell Marque (Rocklin, CA, USA). The antibodies and their respective clones are shown in Table 2.

**Table 2:** Antibodies Used at Creighton University Medical Center- Bergan Mercy Histology Laboratory

Antibody	Clone
Anti CD117	YR145
Anti DOG1	SP31
Anti Desmin	DE-R-11
Anti S100	Polyclonal
Cytokeratin AE1/AE3	PCK26
Low Molecular Weight Cytokeratin	34 βE11
High Molecular Weight Cytokeratin	34 βE12
Anti CD34	QBEnd/10
Anti MelanA	A103

For molecular testing, the mesenteric mass biopsy specimen was sent out to NeoGenomics (Fort Myers, FL, USA) for testing of the *PDGFRα* and *c-KIT *mutation. Per the NeoGenomics report, molecular analysis for *c-KIT *mutation is accomplished when nucleic acid is isolated from formalin-fixed paraffin-embedded tissue. The mutations are tested with the Sanger sequencing bidirectional method and KIT exons 8, 9, 11, 13, and 17 were evaluated. PDGFRα hotspots in exons 12 and 18 were also evaluated using Sanger bidirectional sequencing.

## Discussion

We performed a review of existing literature and identified five patients reported to have GIST with chondroid differentiation. Four patients had GIST with chondroid differentiation prior to imatinib therapy and one patient had GIST with chondroid differentiation after imatinib therapy. The patient with GIST post-imatinib therapy was found to have GIST with chondroid differentiation at the site of metastasis. The patients’ mean age was 64 and ranged from 38 to 79 years old. Four of five patients were female, including the only patient who had chondroid differentiation in metastatic GIST post-imatinib therapy. Table 3 and Table 4 summarize the demographics and pertinent pathology findings for the cases we identified for patients before and after imatinib therapy, respectively.

**Table 3:** Pathologic Findings for Patients With Gastrointestinal Stromal Tumor (GIST) With Chondroid Differentiation Before Imatinib Therapy HPF=High-Power Field

Article	Primary Location/Size	Histomorphology	Immunohistochemistry/Molecular and Cytogenetics	Additional Findings	Age/Sex
Yu et al., 2019 [7]	Lesser curvature of gastric antrum/2.5 x 1.8 x 1.5 cm	Epithelioid cells arranged as chondroid cells in submucosa with myxoid degeneration and hyalinization in background, mitotic count less than 5/50 HPF	Tumor cells: DOG1 (+), CD117 (+), CD34 (+), and INI1 (+), Ki-67 (+) at 2%, S100 (-), SMA (-), Cytokeratin (unspecified type) (-), Desmin (-), HMB45 (-), Synaptophysin (-), NSE (-), EMA (-), MelanA (-). Molecular: *PDGFRα* and *c-kit *mutations evaluated. Asp842Val mutation in PDGFRα 18 exon, negative for mutation in KIT exons 9, 11, 13 and PDGFRα 12 exon	Patient also had poorly differentiated adenocarcinoma of stomach along with the GIST with chondroid differentiation	64/Male
Musaad et al., 2016 [8]	Between upper left pole of kidney and posterior gastric fundus/Size unspecified	Cartilage lobules surrounded by spindle-shaped cells without mitotic figures	Spindle cells: CD117 (+), S100 (+). Cartilage cells: S100 (+), CD117 (-)	The lesion was cystic and multilocular. GIST presented with abscess.	38/Female
Brar et al., 2012 [9]	Originated from greater curvature of stomach, adherent to diaphragm and liver, extended to pylorus /7012 grams	Chondroid matrix with discohesive epithelioid cells; other areas have more spindle-shaped cells with associated necrosis, hemorrhage, and high mitotic activity (65/50 HPF)	Tumor cells: CD117 (+), CD34 (+), CD 99(+), SMA (+), S100 (-). Molecular: Negative for *c-kit *and* PDGFRα* mutations.	Gross appearance that is multinodular with areas of hemorrhage, necrosis, and cystic degeneration. There was a periumbilical mass that was part of the original tumor that was incarcerated in umbilical hernia and detached from original tumor	84/female
Pulcini et al., 2009 [10]	Fundus of stomach/7 x 5 cm	Spindle cell type with whorled pattern that surrounds area of chondroid differentiation	Spindle cells: CD117 (+), S100 (-), NSE (-), CK7(-), CK20(-), CD34 (-), MIB-1 (-), AML (-), AMS (-), chromogranin (-), alpha-1-antitrypsin (-), vimentin (-). Molecular: Negative for mutation in *HER-2/neu*,* EGFR*, and *p16 *gene, strong aneusomy (polysomy in more than two spots) for chromosome 1, normal disomy in chromosomes 7, 8, 9, 17, and 18	MIB-1 index showed a low-grade GIST. Mutation for *c-kit *and *PDGFRα *genes were not assessed.	79/Female

**Table 4:** Pathologic Findings for Patients With Gastrointestinal Stromal Tumor (GIST) With Chondroid Differentiation After Imatinib Therapy

Article	Primary Location/Size	Histomorphology	Immunohistochemistry/Molecular and Cytogenetics	Additional Findings	Age/Sex
Pulvers et al., 2020 [6]	Stomach/130 mm	Primary GIST in stomach: Spindle cell morphology; Mitotic rate 18 per 5 mm^2^, Metastatic GIST to liver: Chondrocytes with mild cytologic atypia among scattered spindle cells in background of myxoid change and hyalinized stroma; cellular component is less than 5% of lesion.	Primary GIST in stomach: CD117 (+), DOG1 (+), subgroup of spindle cells desmin (+), Molecular for primary GIST: KIT exon 11 deletion variant c.1669_1674 deletion with p. (Trp557_Lys558del) variant. Metastatic GIST in liver: Spindle cells: Desmin (+), CD34 (-), CD117(-), and DOG1 (-), Molecular for metastatic GIST: KIT exon 11 deletion variant c.1669_1674 deletion with p. (Trp557_Lys558del) variant.	GIST metastasized to subcapsular region of liver measuring 28 mm; it was found 31 months after the initial resection of primary GIST.	54/Female

GISTs vary in risk of malignancy based on anatomic site, mitotic rate, and size [11,12]. Our patient had high histologic grade both in the primary GIST and recurrent GIST. Furthermore, the size of the tumor and mitotic index of our patient put the patient in a high risk for malignancy stratification [13].

There are multiple reported cases of histomorphologic alterations after imatinib therapy that do not include chondroid differentiation. Pauwels et al. report a case of a 46-year-old male patient with a primary GIST in the stomach with spindle-shaped morphology that subsequently metastasized to various parts of the intra-abdominal region. Hematoxylin and eosin-stained tissue sections showed large epithelioid cells that resemble carcinomatous, melanoma-like and histiocytic proliferation in the duodenum after imatinib therapy [5]. Karakas et al. also reported a case with a 69-year-old male who had spindle and epithelioid type GIST. After imatinib therapy, his GIST was dedifferentiated and had anaplastic features with high mitotic activity and nuclear atypia [14].

GISTs have a wide variety of histomorphologic presentations both before and after imatinib therapy, though few have presented with chondroid differentiation. This article identified four documented incidences of patients with GIST with chondroid differentiation prior to imatinib therapy and one case of GIST with chondroid differentiation after imatinib therapy. The chondroid differentiation after imatinib therapy occurred at the site of metastasis. Therefore, our case of GIST is the first reported case to have chondroid differentiation at the primary recurrence site after imatinib therapy.

## Conclusions

Our case and literature review highlight the importance of identifying the various histomorphologic changes that GIST can undergo before and after imatinib therapy. The case also illustrates the diagnostic challenges pathologists may encounter in evaluating patients with recurrent GIST post-imatinib therapy. Furthermore, this case report and literature review reflects how processes such as chondroid differentiation should be considered for these patients alongside histomorphologic mimickers such as chondrosarcoma.
